# Dietary Practices, Dining Out Behavior, and Physical Activity Correlates of Weight Loss Maintenance

**Published:** 2007-12-15

**Authors:** Judy Kruger, Heidi Michels Blanck, Cathleen Gillespie

**Affiliations:** Physical Activity and Health Branch, Division of Nutrition, Physical Activity, and Obesity, Centers for Disease Control and Prevention; Nutrition Branch, Division of Nutrition, Physical Activity, and Obesity, Centers for Disease Control and Prevention, Atlanta, Georgia; Obesity Prevention and Control Branch, Division of Nutrition, Physical Activity, and Obesity, Centers for Disease Control and Prevention, Atlanta, Georgia

## Abstract

**Introduction:**

Loss of excess weight can improve blood lipids, insulin sensitivity, and blood pressure. However, data are scant on behavioral strategies related to maintenance of weight loss. We examined dietary practices, physical activity, and self-efficacy among adults self-reported to be successful at maintaining weight loss.

**Methods:**

Using the 2004 Styles survey, a mailed survey of U.S. adults aged 18 years or older, we examined behaviors associated with weight loss maintenance among people who reported trying to lose weight. We analyzed data on number of daily fruit and vegetable servings, minutes per week of physical activity, dining out behavior, and confidence in one's ability to engage in behavioral strategies. We conducted frequency and multivariable logistic regression analyses.

**Results:**

More men (35.5%) than women (27.7%) were classified as successful weight loss maintainers. Compared with adults who reported eating at a fast-food restaurant two or more times per week, adults who reported not eating at fast-food restaurants were more successful at weight loss maintenance (odds ratio, 1.62; 95% confidence interval, 1.09–2.42). Compared with adults who consumed fewer than five fruit and vegetable servings per day and were sedentary, adults who consumed fewer than five fruit and vegetable servings per day and accrued 420 minutes or more per week of physical activity or consumed five or more fruit and vegetable servings and accrued 150 minutes or more per week of activity were more successful at weight loss maintenance.

**Conclusion:**

The behavioral strategy of reducing consumption of fast foods could assist people in keeping weight off. The combined approach of consuming five or more fruit and vegetable servings per day and attaining 150 minutes or more per week of physical activity was a common strategy among adults successful at weight loss maintenance.

## Introduction

An increasing number of people worldwide are obese or overweight, and being overweight increases the risk of developing chronic diseases (1). Almost half of adult Americans report that they are trying to lose weight (2,3). Many who lose weight eventually regain most of the lost weight (4-6). Although much research has focused on behaviors that lead to weight loss (7-10), less research is available on weight loss maintenance. Previous work has focused on broader issues (e.g., calories consumed), but data are scant on behavioral strategies related to maintaining weight loss. One widely accepted idea is that successful and sustainable weight loss requires paying attention to both sides of the energy-balance equation: energy intake through food and drink and energy expenditure through physical activity (1). The impact of the combined strategy of eating fruits and vegetables and engaging in regular physical activity has not been widely researched in adults successful at weight loss maintenance.

One of the dietary strategies included in *Dietary Guidelines for Americans 2005* (11) for decreasing energy intake is to eat foods that are low in calories for a given measure of food (i.e., low in energy density). Substituting low-energy–density fruits and vegetables for high-energy–density foods may help decrease overall calorie intake and improve long-term weight loss (12). Another dietary behavior that recently has received attention is the consumption of foods prepared away from home (13). Foods prepared away from home, such as food from fast-food and casual dining restaurants, are generally higher in calories and less healthful than foods prepared at home (14).

Current recommendations encourage people trying to control their weight to increase their energy expenditure by increasing the amount of physical activity performed (1,4,11). Although the level of physical activity recommended to lose weight or prevent weight gain varies, energy expenditure through physical activity is determined largely by the interaction between frequency, duration, and intensity. Recommended amounts of physical activity for weight management are at least 30 minutes of moderate-intensity physical activity on most days of the week (15). Recommendations for weight loss maintenance range from older (2002) guidelines of 60 minutes on most days of the week (16) to more recent (2005) recommendations of 60 to 90 minutes on most days of the week (11).

In addition to changes in diet and physical activity, the National Heart, Lung, and Blood Institute obesity treatment guidelines (15) emphasize behavioral modification. Behavioral modification often involves behavioral strategies that reinforce changes in diet and physical activity; it can include becoming educated about food preparation, label reading, and self-monitoring of diet and physical activity. Many weight control programs incorporate behavioral modification strategies to help people build confidence in their ability to modify their eating and physical activity behaviors (17) because confidence in one's ability to take action and overcome barriers is believed to be an important personal factor in behavior change (18).

By studying the health behaviors of people who have successfully lost weight and kept it off, scientists can develop new guidance for enhancing long-term weight loss maintenance. The National Weight Control Registry (NWCR) is the largest study of adults aged 18 years or older who have maintained long-term weight loss (19,20). This registry consists of U.S. adults who have maintained a weight loss of at least 30 lb (6.6 kg) for at least 1 year (20). Since the early 1990s it has been the major influence in research on weight loss maintenance. Findings from NWCR participants suggest that common behaviors among people who successfully maintain weight loss include eating a low-fat, high-carbohydrate diet; eating breakfast almost every day; frequently self-monitoring weight; and participating in high levels of physical activity (20).

We used a population-based approach to examine behavioral strategies used by people successful at weight loss. We examine racial and ethnic differences in men and women and describe the combined dietary and physical activity behavior among U.S. adults who were attempting weight loss maintenance. We set out to examine whether the combined behavior of eating higher amounts of low-energy–density fruits and vegetables and engaging in regular physical activity is associated with successful weight loss maintenance. In addition, we assessed respondents' dining out behaviors and confidence in their ability to engage in behavioral strategies that support successful weight loss maintenance.

## Methods

### Sample

Data for these analyses came from the 2004 Porter Novelli HealthStyles and ConsumerStyles databases (21) (also referred to as Styles), which are based on the results of three consumer postal mail panel surveys administered in two waves. The purpose of the Styles survey is to examine health attitudes, behavior, and knowledge to inform development of communication and health promotion planning. The mail panel contains approximately 600,000 potential respondents who are recruited to join through a four-page questionnaire. Stratified random sampling of the mail panel was used to generate a list of 10,000 potential respondents for the ConsumerStyles survey, which was fielded during May and June 2004. Most of the survey sample (n = 5500) was stratified according to region, household income, population density, age, and household size to be nationally representative. A low-income/minority supplement (n = 1500) was used to ensure adequate representation of low-income and minority groups. A supplement for households with children (n = 3000) was used to ensure adequate numbers of respondents for a separate study of children called YouthStyles. A small gift of $2 and a sweepstakes entry opportunity (i.e., to win one prize of $1000 or one of five $50 prizes) were offered to encourage respondents to return the ConsumerStyles survey. In 2004, 6207 people completed the ConsumerStyles survey, yielding a response rate of 62%.

During July and August 2004, after the loss of 32 people from the ConsumerStyles panel, the HealthStyles survey was mailed to the remaining 6175 households that had completed the ConsumerStyles survey. Responses to HealthStyles were received from 4345 people, yielding a response rate of 70%. Health and lifestyle data used in our analysis were mainly from the HealthStyles survey, whereas demographic information (e.g., education, annual household income) was obtained from the ConsumerStyles survey. Although the median age of responders to the HealthStyles survey was older than that of nonresponders (46.4 years for responders vs 38.5 years for nonresponders, *P* < .001), the age group distribution of responders did not differ significantly from the 2000 U.S. census distribution.

### Variable definitions

**Weight control behaviors**

Respondents reported their weight history experience in response to the following question: "Overall, what BEST describes your experience with your weight?" Respondents were asked to select one of the following: 1) I've lost weight and have been able to keep it off, 2) I've lost weight but haven't been able to keep it off, 3) I've tried to lose weight but haven't been successful, 4) I've maintained my weight with conscious effort, 5) I've maintained my weight without effort, 6) I've gained weight and haven't tried to lose it, and 7) I pay no attention to my weight. Participants who reported they lost weight and kept it off were defined as *successful weight loss maintainers*; participants who reported either they had lost weight but had not kept it off or had tried unsuccessfully to lose weight were defined as *unsuccessful weight losers*.

**Dietary behaviors**

The Styles survey asked respondents about their consumption of fruits and vegetables: "How many servings of vegetables did you eat or drink yesterday, not counting potatoes?" and "How many servings of fruit did you eat or drink yesterday?" Respondents were asked to include 100% vegetable or fruit juice and fresh, frozen, or canned vegetables or fruit. The upper tertile of consumption was five servings; therefore fruit and vegetable consumption was categorized as fewer than five servings or five or more servings.

**Physical activity levels**

Physical activity behavior was assessed by asking respondents to answer two questions about the frequency and duration of both moderate- and vigorous-intensity physical activities: "During a usual week in the past month, how many days did you do moderate or vigorous physical activities?" and "What is the average number of minutes you spent on these activities each day?" Respondents were prompted that moderate activities referred to activities that cause an increase in breathing or heart rate, such as fast walking, cycling for pleasure, dancing, and yard work, and that vigorous activities referred to activities that cause large increases in breathing, such as running, aerobics, fast bicycling, competitive sports, or heavy yard work. Responses were combined to create categories for total time of weekly activity: none, fewer than 150 minutes, 150 to 419 minutes; 420 to 629 minutes, and 630 minutes or more of moderate- or vigorous-intensity activity.

**Dining out behaviors**

Respondents were asked about the number of nights during the last week they had engaged in certain dining out behaviors. The lead-in to the question was the following: "In the past 7 days, on how many nights did you (or the person who makes dinner in your household): 1) make dinner at home, 2) go out to a fast-food restaurant to eat, 3) go out to a nonfast-food restaurant to eat, 4) bring home take-out food from a restaurant, 5) bring home prepared food from a supermarket, or 6) order food to be delivered to your home?" We created the variable "days per week bring home or have delivered prepared food" by combining "bring home take out," "bring home prepared food from supermarket," and "order delivered food." The following dining behaviors also were combined to create a new index variable, "days per week eat away from home," by combining "eat at fast-food restaurant" and "eat at nonfast-food restaurant." Respondents were asked to indicate the average number of times per week they made dinner at home using a number from zero through seven. Responses were classified into the following: less than three, three to five, and six to seven times per week. With a separate question, respondents were asked, "Which of the following would you say you often do when eating out at a restaurant?" Participants were asked to respond either yes or no to all that apply: 1) order an appetizer to serve as an entrée, 2) split an entrée with someone, 3) order a half-portion of an entrée, and 4) split a dessert with someone. The first three were combined to create "order reduced entrée or split an entrée."

**Behavioral strategies**

Respondents were asked to rate their level of confidence in their ability to engage in certain dietary behaviors on a scale of 1 through 10. The questions centered on the following behaviors: "Keep track of the number of calories you eat," "Eat smaller amounts of food at each meal to control or lose weight," "Balance the amount of food you eat each day with how active you are," "Keep fewer high-fat or high-calorie snack foods in your house," "Snack on fruits and vegetables instead of high-calorie or high-fat snacks," "Limit dining out (e.g., restaurant, fast food, pizza, sandwich shop, or take-out) to only two times a week." The responses were grouped into the following three categories: not confident (response of 1–3), somewhat confident (response of 4–7) and very confident (response of 8–10). Missing responses were not included in the analyses.

### Statistical analysis

From the 4345 HealthStyles respondents, we first limited our analytic sample to the 2124 participants whom we classified as successful weight loss maintainers (n = 587, 14.4% [weighted]) or unsuccessful weight loss maintainers (n = 1537, 32.1% [weighted]). Characteristics (e.g., age, race/ethnicity, education, income) of respondents included in the analyses were similar to those of respondents not included, with the exception of sex. However, we examined weight loss maintenance among men and women separately. From the 4345 respondents, we excluded the following 2221: respondents who had maintained weight with effort (n = 765) or without effort (n = 598); respondents who gained weight and had not tried to lose it (n = 355); respondents who paid no attention to weight (n = 304); and respondents who were missing data on weight loss or maintenance (n = 199). Of the remaining 2124, we then excluded respondents with missing self-reported height or weight (n = 77); respondents who reported extreme height or weight values (outside the 1st–99th percentile of measured height or weight values in the 1999–2002 National Health and Nutrition Examination Survey) (n = 44); female respondents who stated they were currently pregnant or who did not respond to the question on pregnancy (n = 52); and respondents who were missing data on moderate- or vigorous-intensity activity (n = 260) or fruit and vegetable intake (n = 17). Some participants met one or more exclusion criteria. After exclusions, the final sample numbered 1713, with 648 men and 1065 women.

We calculated the prevalence of respondents who were successful at maintaining weight according to sex, age, race/ethnicity, education, income, body mass index (BMI [kg/m^2^]), fruit and vegetable servings, physical activity level, dining out behaviors, and confidence in their ability to engage in specific behavioral strategies. We used multivariable logistic regression to calculate adjusted odds ratios (ORs) with 95% confidence intervals (CIs) for those successful (versus unsuccessful) at weight loss maintenance. We excluded from the individual comparisons data that were missing because of participant nonresponse. The data were poststratified and weighted to the U.S. census population on age, race/ethnicity, sex, household size, and household income to create a population-based data file. We conducted all analyses using SAS version 9.1-callable (SAS Institute Inc, Cary, NC) and SUDAAN version 9.0 (Research Triangle Institute, Research Triangle Park, NC) software to account for the complex sampling design and weighting procedure.

## Results

Among adults trying to lose weight, 35.5% of men (Table 1) and 27.7% of women (Table 2) were successful weight loss maintainers. Sex-specific regression models showed that men were less likely to maintain weight loss if they were overweight or obese than if they were of normal weight (Table 1). Men who engaged in physical activity 420 to 629 minutes per week or 630 minutes per week or more were more likely to maintain weight loss than were those who were sedentary.

Among women successful at weight loss maintenance, black women were more likely to maintain weight loss than were white women (Table 2). Women were less likely to maintain weight loss if they were overweight or obese than if they were of normal weight. Women who consumed five or more fruit and vegetable servings on the previous day were more likely to maintain weight loss than women who consumed fewer than five fruit and vegetable servings on the previous day. Women who engaged in 150 to 629 minutes per week of physical activity (equivalent to 30 to 90 minutes per day) were more likely to maintain weight loss than women who were sedentary. Specifically, women were more likely to maintain weight loss if they engaged in 150 to 419 minutes per week of physical activity or 420 to 629 minutes per week than were women who were sedentary. Women who engaged in the highest level of physical activity (≥630 minutes per week) were not significantly more likely to maintain weight loss than women who were sedentary.

Among men and women who consumed fewer than five fruit and vegetable servings on the previous day, people who exercised the most (≥420 minutes per week) were more likely to maintain weight loss than people who were sedentary (Table 3). Compared with men and women who consumed fewer than five fruit and vegetable servings on the previous day and were sedentary, participants who consumed five or more fruit and vegetable servings on the previous day and engaged in physical activity 150 to 419 minutes per week or 420 minutes or more per week were more likely to maintain weight loss.

After adjusting for sex, race/ethnicity, education, income, BMI, and physical activity, we found similar odds of successful weight loss maintenance for people who often ordered a reduced-size entrée when dining out and people who ordered regular-size entrées. Adults who did not eat at fast-food restaurants were more likely to maintain weight loss than people who reported fast-food dining two or more times per week (Table 4).

Analysis of confidence in one's ability to engage in dietary strategies showed that respondents who were more confident in their ability to engage in certain behaviors were more successful at weight loss maintenance than those who were not confident (Table 5). Specifically, adults who reported being very confident in their ability to engage in certain behavioral strategies (i.e., keep track of calories consumed, eat smaller amounts at each meal, balance amount of food with activity level, keep fewer high-fat, high-calorie snacks at home, snack on fruits and vegetables instead of high-fat or high-calorie snacks, and limit dining out to two times per week) had adjusted odds of being successful at weight loss maintenance that were 57% to 229% higher than those who reported no confidence.

## Discussion

Although studies have linked the consumption of fruits and vegetables and regular physical activity to the management of chronic diseases (16), no epidemiologic studies have focused on the combined prevalence of fruit and vegetable consumption and physical activity among people engaging in weight loss and maintenance. Findings from our population-based survey suggest that higher levels of weekly physical activity were needed for successful weight loss maintenance if the respondent consumed fewer than five low-energy–density fruit and vegetable servings on the previous day. Our data provide insights into the details of behavioral patterns among people reporting success at weight loss maintenance and support findings in the literature that suggest both dietary and physical activity approaches are key in helping people manage their weight (1,11,13).

One common characteristic among people who were successful at weight loss maintenance is their participation in regular physical activity. These results are consistent with past research documenting the importance of physical activity in successful weight loss maintenance (22). The highest odds for being successful at weight loss maintenance among men and women were among those who reported high levels of physical activity (approximately 420–630 minutes per week). Time spent engaged in physical activity can allow people to increase their energy intake and may assist adults in maintenance of weight loss (1). The total amount of energy expended with each activity session depends on the intensity, frequency, and duration of activity and is a function of respondents' body weight and fitness level (23).

In this study, men and women successful at weight loss maintenance reported different individual behaviors. Among women who reported consuming five or more fruit and vegetable servings on the previous day, one-third were successful at weight loss maintenance. Among women who reported consuming fewer than five fruit and vegetable servings, one-fourth were successful. However, we found higher odds of successful weight loss maintenance among adults who engaged in the combined behaviors of eating five or more fruit and vegetable servings per day and moderate to high levels of physical activity. Data from the NWCR also found that participants who have maintained long-term weight loss reported that fruits and vegetables made up a large percentage of food items reported on a food frequency questionnaire (24). Substituting low-energy–density foods (e.g., broth-based soups, grains, fruit, and vegetables) for high-energy–density foods may increase the feeling of fullness and help reduce energy intake, thereby assisting with weight loss maintenance (13).

Data on consumption of foods away from home suggest that when dining out, people eat more food, higher-calorie food, or both (25). Therefore, dining behavior is a potentially modifiable contributor to caloric intake and weight control. If adults are dining outside of the home at limited-service, mid-scale full service, or casual dining full-service restaurants, they are less likely to prepare or consume food at home (26). Away-from-home foods purchased from limited-service restaurants are typically high in fat and calories (13). Our finding that a higher proportion of people (34.0%) who ordered reduced-size entrées when dining out were more successful at weight loss maintenance than the proportion (28.0%) who ordered regular-size entrées may be partially explained by portion size. Research shows that people consume more calories when presented with larger portions (27) and that food eaten away from home is higher in calories and fat than food consumed at home. For example, data from 1995 show that foods consumed at home have an average of 31.5% of calories from fat, compared with 37.6% of calories from fat for foods consumed away from home (25). Elfhag and Rossner (28) show in their review that successful weight loss maintenance was associated with lower total caloric intake, reduced portion sizes, reduced frequency of snacks, and less dietary fat; dining behavior was not specifically mentioned. We found higher odds of success at weight loss maintenance among people who reported sharing portions and among those who reported never eating at fast-food restaurants, compared with people who eat at fast-food restaurants two or more times per week. In the Pound of Prevention study, the increase in number of visits to fast-food restaurants was associated with lower dietary restraint (29). Our findings suggest that people successful at weight loss maintenance have adopted the behavior of consuming smaller portion sizes by sharing food or eating a reduced amount (e.g., half-size, appetizer size) or by infrequently, if ever, dining at fast-food restaurants.

In our study, respondents' level of confidence in their ability to engage in diet modification, including eating smaller amounts of food, balancing food intake with activity, and keeping track of calories, was also related to successful weight loss maintenance. Normative beliefs, such as confidence, can act as a motivating factor for behavior change (30). In a comprehensive review by Teixeira et al (31), psychosocial constructs such as self-efficacy related to diet and exercise were important for successful weight management. Strategies used in weight loss and weight management programs include stimulus control by setting incremental goals (i.e., reducing the number of visits per week to fast-food restaurants), self-monitoring of eating habits and physical activity (i.e., objectively documenting one's own behavior through observation and recording), and contingency management (i.e., use of rewards for specific actions). The aim of these techniques is to alter eating and activity habits over the long term (15). More research about how people make behavioral choices can play an important role in weight management and may help adults gain confidence in their ability to modify dietary and physical activity behaviors, which can lead to long-term healthy lifestyles.

Our analysis is subject to several limitations. First, Styles participants are obtained through survey panels, which commonly are used in marketing research but less commonly in health research. Research comparing findings from paneling techniques and traditional health-research sampling techniques has found similar prevalence responses to several survey items (21). However, the Styles survey is based on self-reported data and thus contains all the limitations inherent in self-report. Second, data from the Styles survey are cross-sectional, and the temporal sequence of behaviors and successful weight loss maintenance cannot be determined. Third, the questionnaire did not determine how much weight was lost. Although consensus does not exist about how to define successful weight loss maintenance, knowing how much weight respondents lost and how long they kept it off is important (32). Fourth, the questionnaire did not include an in-depth dietary assessment, which limits analysis of details about food consumption patterns. The fruit and vegetable questions have not been validated or tested for reliability and represent only a single day of consumption, which may not be representative of typical consumption. Although no cognitive testing was performed and the items were not formally validated, the survey questions used were vetted with experts and pilot tested for clarity. Similar physical activity questions have been subject to reliability and validity testing; it seems likely that the questions used in our study would show similar levels of validity (33). Moreover, the questionnaire asked about dining behaviors in terms of the number of nights in the last week, which was used as a proxy for a usual week. Fifth, despite oversampling, our analytic sample included only a small number of minority male participants, and respondents were highly educated, which may limit overall generalizability.  

Our study suggests that one dietary strategy associated with successful weight loss maintenance was eating infrequently at fast-food restaurants. The combined approach of consuming five or more fruit and vegetable servings on the previous day and accruing 150 minutes or more per week of physical activity also was associated with successful weight loss maintenance. Further research is needed to determine an array of practical dietary strategies and modes of physical activity that help people develop long-term healthful habits that can result in improved health and quality of life through successful weight loss maintenance.

## Figures and Tables

**Table 1:** Prevalence and Odds^a^ of Successful Weight Loss Maintenance^b^ Among Men, Styles Survey,^c^ 2004

Characteristic	All Male Respondents		Male Respondents Successful at Weight Loss Maintenance	

	N	% (95% CI)	% (95% CI)	OR (95% CI)
**Total**	648	NA	35.5 (31.0-40.2)	NA
**Age group, y**					
18-29	34	12.8 (8.6-18.5)	48.3 (28.3-68.9)	Referent group
30-44	230	33.6 (29.6-37.9)	29.9 (23.7-36.9)	0.59 (0.23-1.55)
45-64	278	36.8 (32.8-41.1)	28.3 (22.9-34.3)	0.57 (0.22-1.47)
≥65	106	16.8 (13.8-20.1)	52.7 (42.7-62.4)	1.44 (0.54-3.89)
**Race/ethnicity**					
White	489	74.2 (69.7-78.3)	33.1 (28.3-38.3)	Referent group
Black or African American	43	6.7 (4.8-9.2)	40.8 (25.2-58.4)	1.29 (0.59-2.83)
Hispanic or Latino	72	12.0 (9.1-15.6)	36.5 (23.2-52.1)	1.19 (0.60-2.35)
Other	44	7.1 (4.7-10.6)	53.5 (33.7-72.2)	1.39 (0.60-3.24)
**Education**					
≤High school graduate	184	23.2 (19.9-26.8)	27.3 (20.8-34.9)	0.61 (0.33-1.11)
Some college	203	30.6 (26.6-35.0)	31.6 (24.2-40.0 )	0.78 (0.46-1.33)
College graduate	203	31.6 (27.6-35.9)	40.1 (32.9-47.8)	Referent group
Missing data	58	14.6 (10.8-19.4)	46.6 (30.7-63.2)	1.37 (0.60-3.14)
**Annual income, $**					
<25,000	158	22.3 (18.4-26.7)	41.5 (30.7-53.3 )	1.09 (0.56-2.10)
25,000-60,000	206	33.9 (29.5-38.5)	32.4 (24.8-41.2)	1.00 (0.58-1.70)
>60,000	284	43.9 (39.5-48.4)	34.7 (29.0-40.8)	Referent group
**Body mass index**					
<25.0	112	18.2 (14.8-22.2)	65.2 (54.5-74.6)	Referent group
25.0-30.0	243	39.2 (34.7-43.9)	39.1 (31.4-47.4)	0.38 (0.22-0.66)
>30.0	293	42.5 (38.1-47.1)	19.3 (14.6-25.1)	0.15 (0.09-0.28)
**No. of fruit and vegetable servings per day**					
<5	396	61.3 (56.6-65.7)	32.5 (27.0-38.6)	Referent group
≥5	252	38.7 (34.3-43.4)	40.1 (32.7-47.9)	1.04 (0.67-1.60)
**Minutes per week of moderate- or vigorous-intensity physical activity**					
None	78	11.9 (9.2-15.2)	22.4 (13.7-34.5)	Referent group
1-149	235	36.5 (32.2-41.1)	24.9 (18.1-33.3)	0.98 (0.49-1.97)
150-419	205	31.2 (27.0-35.7)	42.1 (33.6-51.0)	1.89 (0.95-3.78)
420-629	60	9.0 (6.8-11.7)	54.7 (41.0-67.8)	3.77 (1.60-8.88)
≥630	70	11.4 (8.9-14.6)	49.5 (36.5-62.5)	2.53 (1.04-6.19)

Ns are unweighted; percentages, weighted. CI indicates confidence interval; OR, odds ratio; NA, not applicable.

a Odds for successful weight loss maintenance versus unsuccessful are adjusted for age, race/ethnicity, education, income, and body mass index.

b Successful weight loss maintenance refers to participants who reported they lost weight and have kept it off.

c Porter Novelli HealthStyles and ConsumerStyles databases (21).

**Table 2:** Prevalence and Odds^a^ of Successful Weight Loss Maintenance^b^ Among Women, Styles Survey,^c^ 2004

Characteristic	All Female Respondents		Female Respondents Successful at Weight Loss Maintenance	

	N	% (95% CI)	% (95% CI)	OR (95% CI)
**Total**	1065	NA	27.7 (24.3-31.4)	NA
**Age group, y**				
18-29	89	19.2 (15.3-23.8)	35.6 (23.7-49.5)	Referent group
30-44	427	35.2 (32.0-38.6)	23.3 (19.2-27.9)	0.52 (0.25-1.09)
45-64	420	32.3 (29.3-35.5)	23.6 (19.6-28.2)	0.62 (0.31-1.27)
>65	129	13.3 (11.1-15.7)	38.0 (29.5-47.2)	1.36 (0.65-2.85)
**Race/ethnicity**				
White	758	71.3 (67.8-74.6)	26.0 (22.2-30.2)	Referent group
Black or African American	130	12.1 (9.9-14.6)	32.5 (23.5-43.1)	1.96 (1.10-3.49)
Hispanic or Latino	124	12.4 (10.0-15.3)	33.9 (22.7-47.2)	1.45 (0.83-2.54)
Other	53	4.2 (3.0-5.9)	24.4 (12.5-42.4)	0.66 (0.28-1.57)
**Education**				
≤High school graduate	309	28.6 (25.3-32.1)	28.0 (22.0-34.9)	1.02 (0.57-1.82)
Some college	428	39.4 (35.9-43.1)	25.3 (20.6-34.9)	0.82 (0.50-1.33)
College graduate	324	31.8 (28.4-35.4)	30.6 (24.1-38.1)	Referent group
Missing data	4	0.2 (0.1-0.6)	0.0 (0.0)	NA
**Annual income, $**				
<25,000	303	27.3 (24.0-30.9)	28.9 (22.2-36.6)	0.94 (0.57-1.54)
25,000-60,000	360	38.0 (34.3-41.8)	29.1 (23.0-36.0)	1.26 (0.83-1.92)
>60,000	402	34.7 (31.5-38.0)	25.3 (21.0-30.1)	Referent group
**Body mass index**				
<25.0	236	22.7 (19.7-26.0)	56.1 (48.4-63.6)	Referent group
25.0-30.0	340	32.4 (29.1-36.0)	24.6 (19.4-30.5)	0.23 (0.15-0.35)
>30.0	489	44.8 (44.2-48.6)	15.6 (11.3-21.1)	0.13 (0.08-0.21)
**No. of fruit and vegetable servings per day**				
<5	639	61.6 (58.0-65.1)	24.4 (20.3-29.1)	Referent group
≥5	426	38.4 (34.9-42.0)	33.0 (27.4-39.0)	1.60 (1.08-2.38)
**Minutes per week of moderate- or vigorous-intensity physical activity**				
None	160	15.2 (12.6-18.1)	20.5 (14.4-28.4)	Referent group
1-149	435	39.3 (35.8-43.0)	21.2 (16.7-26.5)	0.96 (0.56-1.66)
150-419	341	32.5 (29.1-36.1)	33.3 (27.1-40.0)	1.92 (1.08-3.40)
420-629	64	6.9 (4.9-9.6)	49.7 (33.0-66.5)	2.91 (1.42-5.94)
≥630	65	6.1 (4.6-8.0)	33.0 (21.7-46.7)	1.57 (0.66-3.75)

Ns are unweighted; percentages, weighted. CI indicates confidence interval; OR, odds ratio; NA, not applicable.

a Odds of successful weight loss maintenance versus unsuccessful are adjusted for age, race/ethnicity, education, income, and body mass index.

b Successful weight loss maintenance refers to participants who reported they lost weight and have kept it off.

c Porter Novelli HealthStyles and ConsumerStyles databases (21).

**Table 3:** Prevalence and Odds^a^ of Successful Weight Loss Maintenance^b^, by Fruit and Vegetable Intake and Level of Moderate- or Vigorous-Intensity Physical Activity, Styles Survey,^c^ 2004

Minutes per Week of Physical Activity	All Respondents		Respondents Successful at Weight Loss Maintenance	

	N	% (95% CI)	% (95% CI)	OR (95% CI)
**Fewer than five fruits and vegetables yesterday**				
None	163	9.3 (7.7-11.1)	21.4 (15.2-29.3)	Referent group
1-149	424	24.7 (22.3-27.2)	20.7 (16.3-52.9)	0.80 (0.47-1.35)
150-419	310	18.8 (16.6-21.3)	33.2 (26.6-40.6)	1.36 (0.80-2.29)
≥420	138	8.6 (7.1-10.5)	43.7 (33.4-54.5)	2.03 (1.12-3.68)
**Five or more fruits and vegetables yesterday**				
None	75	4.5 (3.4. 5.9)	20.9 (12.3-33.3)	0.66 (0.31-1.42)
1-149	246	13.4 (11.6-15.5)	26.4 (19.1-35.4)	0.94 (0.53-1.66)
150-419	236	13.1 (11.3-15.1)	42.2 (34.5-50.4)	2.07 (1.17-3.67)
≥420	121	7.5 (6.2-9.2)	51.3 (41.0-61.4)	2.39 (1.24-4.60)

Ns are unweighted; percentages, weighted. CI indicates confidence interval; OR, odds ratio.

a Odds of successful weight loss maintenance versus unsuccessful are adjusted for sex, age, race/ethnicity, income, and body mass index. Each predictor variable is examined separately.

b Successful weight loss maintenance refers to participants who reported they lost weight and have kept it off.

c Porter Novelli HealthStyles and ConsumerStyles databases (21).

**Table 4:** Prevalence and Odds^a^ of Successful Weight Loss Maintenance^b^, by Dining Out Behaviors in the Past Week,  Styles Survey,^c^ 2004

Behavior	All Respondents		Respondents Successful at Weight Loss Maintenance	

	N	% (95% CI)	% (95% CI)	OR (95% CI)
**Often order reduced-size entrée^d^**					
Yes	903	50.8 (47.9-53.7)	34.0 (30.0-38.2)	1.28 (0.96-1.71)
No	810	49.2 (46.3-52.1)	28.0 (24.2-32.1)	Referent group
**Often split a dessert**					
Yes	635	38.7 (35.9-41.6)	33.2 (28.7-38.0)	1.12 (0.84-1.50)
No	1078	61.3 (58.4-64.1)	29.6 (26.1-33.4)	Referent group
**Days per week make dinner at home^e^**					
<3	191	13.1 (11.1-15.4)	29.5 (21.7-38.8)	Referent group
3-5	865	51.3 (48.4-54.2)	28.5 (24.6-32.7)	1.02 (0.63-1.64)
6-7	645	35.0 (32.4-37.8)	35.2 (30.8-39.8)	1.28 (0.80-2.05)
**Days per week eat away from home^e^,^f^**					
None	460	25.0 (22.7-27.5)	31.8 (27.0-37.0)	1.30 (0.64-2.68)
1	322	18.1 (16.0-20.3)	32.2 (26.0-39.1)	1.27 (0.61-2.65)
2	345	21.1 (18.7-23.8)	34.4 (27.3-42.2)	1.64 (0.75-3.55)
3-4	252	15.6 (13.6-18.0)	27.1 (20.7-34.7)	1.00 (0.46-2.15)
≥5	82	5.0 (3.9-6.4)	28.2 (18.3-40.7)	Referent group
**Days per week eat at fast-food restaurant^e^**					
None	756	44.1 (41.2-47.0)	36.9 (32.5-41.5)	1.62 (1.09-2.42)
1	432	25.0 (22.6-27.5)	26.6 (21.4-32.6)	1.03 (0.66-1.63)
≥2	366	21.6 (19.2-24.1)	25.8 (20.5-31.9)	Referent group
**Days per week eat at nonfast-food restaurant^e^**					
None	724	39.0 (36.2-41.8)	29.0 (25.3-33.0)	0.78 (0.51-1.21)
1	528	31.3 (28.6-34.1)	31.7 (26.5-37.5)	0.87 (0.55-1.38)
≥2	289	19.4 (17.1-22.0)	36.1 (29.0-43.8)	Referent group
**Days per week bring home or have delivered prepared food^e^,^g^**					
None	705	41.0 (38.2-43.9)	37.1 (32.7-41.8)	1.45 (0.93-2.27)
1	337	18.5 (16.4-20.8)	26.9 (21.0-33.7)	1.17 (0.69-1.95)
≥2	326	19.5 (17.1-22.1)	26.2 (19.9-33.7)	Referent group
**Days per week bring home take-out^e^**					
None	930	54.0 (51.1-56.9)	34.6 (30.7-38.8)	1.10 (0.70-1.74)
1	398	22.8 (20.5-25.3)	23.6 (18.6-29.5)	0.70 (0.41-1.21)
≥2	180	10.2 (8.7-12.1)	30.5 (22.3-40.2)	Referent group
**Days per week bring home prepared food from supermarket^e^**					
None	1088	62.0 (59.1-64.9)	33.4 (30.0-37.1)	1.52 (0.84-2.75)
1	231	13.9 (11.9-16.3)	26.5 (18.3-36.7)	1.14 (0.53-2.46)
≥2	126	7.6 (6.1-9.5)	25.2 (15.9-37.6)	Referent group
**Days per week order delivery food^e^**					
None	1187	68.8 (66.0-71.4)	32.8 (29.4-36.5)	1.32 (0.61-2.85)
1	199	11.8 (9.9-13.9)	22.3 (15.2-31.4)	0.92 (0.39-2.18)
≥2	66	3.9 (2.9-5.4)	30.2 (17.7-46.5)	Referent group

Ns are unweighted; percentages, weighted. CI indicates confidence interval; OR, odds ratio.

a Odds of successful weight loss maintenance versus unsuccessful are adjusted for sex, age, race/ethnicity, education, income, body mass index, and physical activity. Each predictor variable is examined separately.

b Successful weight loss maintenance refers to participants who reported they lost weight and have kept it off.

c Porter Novelli HealthStyles and ConsumerStyles databases (21).

d Combines split entrée, half-portion, or appetizer as entrée responses.

e Ns do not total 1713 within each category because not all respondents answered all questions.

f Combines fast food or nonfast-food restaurant as responses.

g Combines take-out, prepared from supermarket, or delivered responses.

**Table 5:** Prevalence and Odds^a^ of Successful Weight Loss Maintenance^b^, by Participants' Confidence^c^ in Their Ability to Engage in Selected Behavioral Strategies, Styles Survey,^d^ 2004

Behavioral Strategy	All Respondents^e^		Respondents Successful at Weight Loss Maintenance	

	N	% (95% CI)	% (95% CI)	OR (95% CI)
**Keep track of the calories you eat**					
Not confident	773	44.0 (41.1-46.9)	27.7 (23.8-32.0)	Referent group
Somewhat confident	536	32.0 (29.3-34.7)	27.9 (23.1-33.3)	0.94 (0.67-1.33)
Very confident	388	23.4 (20.9-26.0)	40.9 (34.8-47.3)	1.89 (1.32-2.70)
**Eat smaller amounts at each meal**					
Not confident	186	10.9 (9.3-12.8)	16.5 (11.5-23.0)	Referent group
Somewhat confident	772	45.8 (42.9-48.7)	24.4 (20.4-29.0)	1.33 (0.80-2.22)
Very confident	738	42.4 (39.6-45.3)	41.6 (37.3-46.1)	3.29 (2.02-5.36)
**Balance amount of food with activity level**					
Not confident	323	18.8 (16.6-21.1)	20.5 (15.0-27.2)	Referent group
Somewhat confident	868	51.1 (48.2-54.0)	27.0 (23.1-31.4)	1.32 (0.87-1.99)
Very confident	504	29.0 (26.5-31.7)	44.0 (39.0-49.2)	2.49 (1.63-3.83)
**Keep fewer high-fat, high-calorie snack foods at home**					
Not confident	239	13.8 (12.0-15.9)	25.6 (19.8-32.6)	Referent group
Somewhat confident	672	37.6 (34.8-40.5)	24.9 (20.4-30.0)	0.92 (0.59-1.44)
Very confident	782	47.2 (44.3-50.1)	36.9 (32.8-41.2)	1.57 (1.02-2.41)
**Snack on fruits and vegetables instead of high-fat, high-calorie snacks**					
Not confident	167	10.4 (8.8-12.3)	24.3 (17.5-32.6)	Referent group
Somewhat confident	627	35.9 (33.1-38.7)	24.4 (19.9-29.6)	1.10 (0.66-1.85)
Very confident	900	52.7 (49.8-55.6)	36.5 (32.7-40.6)	1.76 (1.08-2.87)
**Limit dining out to two times per week**					
Not confident	161	9.6 (8.1-11.3)	24.7 (17.8-33.3)	Referent group
Somewhat confident	423	26.5 (23.8-29.3)	23.2 (17.6-30.0)	0.84 (0.48-1.48)
Very confident	1109	62.9 (60.0-65.7)	34.8 (31.4-38.4)	1.60 (0.98-2.62)

Ns are unweighted; percentages, weighted. OR indicates odds ratio; CI, confidence interval.

a Odds of successful weight loss maintenance versus unsuccessful are adjusted for age, race/ethnicity, education, income, body mass index, and physical activity. Each predictor variable is examined separately.

b Successful weight loss maintenance refers to participants who reported that they lost weight and have kept it off.

c Responses were provided on a scale of 1 to 10 and were grouped into the following three categories: not confident (response of 1–3), somewhat confident (response of 4–7), and very confident (response of 8–10).

d Porter Novelli HealthStyles and ConsumerStyles databases (21).

e Ns do not total 1713 within each category because not all respondents answered all questions.
